# Focus on vegetable crops

**DOI:** 10.1093/plphys/kiae246

**Published:** 2024-05-01

**Authors:** Qiaohong Duan, Yann-rong Lin

**Affiliations:** College of Horticulture Science and Engineering, Shandong Agricultural University, Tai’an, Shandong 271018, China; Department of Agronomy, National Taiwan University, Taipei 10617, Taiwan; World Vegetable Center, Headquarters, Shanhua, Tainan 74151, Taiwan

Vegetable crops encompass over 200 plant species utilized as human food. These predominantly herbaceous plants can be consumed raw or cooked, either fresh or preserved, in various forms including leaves, roots, fruits, seeds, flowers, inflorescences, and stems. In addition to providing calories, vegetables also provide various micronutrients for healthy diets to combat hidden hunger. Thus, vegetables together with fruits and legumes are proposed as new generation staple food.

Vegetable crops have been domesticated from their wild progenitors that have undergone a process influenced by human preferences and diverse environment factors. Consequently, current vegetable germplasms exhibit relatively high genetic diversity. Because edible parts (organs) of vegetable crops are quite different for each crop, goals in physiological research and breeding are quite different for each crop. Vegetables, like other crops, have faced threats from abiotic and biotic stresses such as global climate change, decreasing arable land, and soil erosion. Overcoming these challenges requires a deep understanding of the fundamental biology and genetics of the crops. Additionally, vegetable breeding needs to consider consumers’ preference and market demands. Integrating high throughput phenotyping data and artificial intelligence analyses will enable smart agriculture in vegetable industry. Therefore, basic research in plant physiology and genetics can provide guidance for cultivation practice and breeding of vegetable crops, achieving sustainable agriculture and increasing food security.

In this Focus Issue on Vegetable Crops, we present a collection of research articles and update reviews that cover the latest research on vegetable crops and highlight the current trends and future directions. The advancements reported in the Focus Issue not only help us better understand the molecular mechanisms underlying growth and development of vegetable crops and their responses to biotic and abiotic stresses but also offer strategies for the vegetable industry to adapt to consumer demands and the changing environments. Here we highlight some of the contents of 2 updates and 21 research articles in this Focus Issues, as well as some in our related Focus Collection.

## From domestication to modern breeding

Crop domestication begins with humans selecting individuals from wild populations based on their preferred traits, gradually transforming them from wild species into cultivated species over thousands or hundreds of years. The prominent traits distinguish cultivated species from wild relatives are considered as domestication syndromes. As humans migrate, these crops consequently spread to different geographic regions and they have adapted to the local agro-ecological environments. They have undergone further selection to meet various diet preferences. Therefore, the complex inter-connection between environmental adaptation through natural selection/cultivation and artificial selection by humans has resulted in not only phenotypic variations but also changes in intrinsic genomic compositions. Genomic studies have led to better understanding of the genetic changes that have occurred during evolution and domestication of the vegetable crops. Genomic data made it feasible to investigate the causal relationships between important horticultural traits and genetic variations (Chen et al. 2024a; Wang et al. 2024e). These studies have identified genes responsible for important traits, further facilitating basic research at biochemical, molecular, and physiological levels and providing new breeding strategies, particularly genomics-assisted breeding and precision breeding. Wang et al. (2024e) used tomatoes, an important fruit vegetable, as an example to illustrate how these results and approaches can be used to improve other crops as well.

Compared with cereal and legume crops, domestication of vegetable crops is relatively more complex because different organs of vegetable crops are consumed and processed quite differently. Chen et al. (2024a) provided a comprehensive review on how selection of domestication syndrome traits and other traits have shaped genetic architectures at both single gene and genomic levels. Some traits such as non-shattering seeds/pods are under strong selection while other traits including fruit size, color, flavor, and dormancy are under moderate selection. Models including hard sweep model, soft sweep model, and polygenic model have been proposed to understand genetic changes in the corresponding genes and other related genomic regions. When crops spread to different agro-ecological regions mediated by human activities, divergent or directional selection on some target genes occur so that crops can adapt to local environment and meet human's preference on cultivation practices and consumption. Popular approaches of genome scan in cultivars can only detect the hard selective sweep, which happened under the restricted conditions requiring a new mutation on a Mendelian gene controlling a trait under directional selection.

Tomato (*Solanum lycopersicum* L.) is recognized worldwide as an essential vegetable crop due to the abundant nutrients such as lycopene, flavonoids, vitamins, and minerals. The global tomato production reached 20 trillion tons in 2022 according to the Food and Agriculture Organization. Tomato has become one of the important model crops for research and breeding. The first tomato genome was published by the Tomato Genome Consortium (2012), paving the way for subsequent updates for high quality genome database, and the latest version, SL5.0 genome, was released by Zhou et al. (2022). These genomic resources accelerated research in tomato multi-omics and breeding. Furthermore, the vast genetic diversity in tomato germplasm, evident from its diversity in fruit size, shape, and color, provides rich genetic resources not only for tomato industry but also for research and breeding applications. The UPDATE article on tomato research by Wang et al. (2024e) offers the latest research in tomato and subsequent applications, providing an excellent template for studying other Solanaceae crops such as peppers. Apart from cultivated tomatoes, there are 12 wild tomato species that offer additional genetic resources for research on tomato domestication and breeding. A total of 66 tomato genomes have been sequenced, enabling tomato pan-genome studies that can reveal key domestication events and unveil genetic variation in SNPs and structural variations associated with important horticultural traits. In addition to implementing marker-assisted selection for the beneficial alleles and genomic selection to accelerate tomato breeding efficiency, Wang et al. (2024e) also reviewed the applications of de novo domestication, male-sterility system, haploid induction system, synthetic biology, and heterosis.

Genetic studies and crop breeding often require inbred lines or pure/homozygous lines. However, it takes 6 to 8 generations to achieve homozygosity starting from a heterozygous F_1_ plant. Furthermore, it is even more challenging for outcrossing plants such as *Cucurbita* vegetables or self-incompatible plants such as *Brassica* vegetables. One quick way to achieve homozygosity is to double a haploid plant, named double-haploid. Developing haploid induction systems is the key step for obtain double-haploid lines. Anther and microspore cultures have been used to produce haploid plants for many plant species, but the efficiency is relatively low. Haploid inducer lines have been identified in natural germplasm of *Arabidopsis* and maize. On the other hand, mutating relevant genes such as *MATRILINEAL* (*MTL*)/*PHOSPHOLIPASE A1* (*PLA1*)/*NOT LIKE DAD* (*NLD*), *CEMTROMERIC HISTONE3* (*CENH3*), and *DOMAIN OF UNKNOWN FUNCTION 679 MEMBRANCE PROTEIN* (*DMP*) to induce haploid formation has been explored. Yin et al. (2024) used CRISPR-Cas9 technology to edit *CsDMP* in cucumber (*Cucumis sativus* L.). The resulting *csdmp* mutants could induce maternal haploids in cucumber, with the average haploid induction rate ranged from 0.09% to 0.04%, providing a new haploid inducer line in cucumber.

## Horticultural traits for consumption

The quality of vegetable products, including appearance quality such as color, shape and size, and intrinsic quality such as nutritional value and safety, greatly influences the market value of vegetables. Fruit shape is an important agronomic trait related to yield, quality, and customer preference. Tian et al. (2024) reported that fruit-related long noncoding RNAs negatively regulate pumpkin fruit development, possibly through interacting with S-adenosyl-L-methionine synthetase (SAMS) to affect ethylene synthesis. In cucumber, fruit neck length varies greatly among different cucumber varieties, but the underlying regulatory mechanisms are poorly understood. Wang et al. (2024a) found that the transcription factor CsMYB36 positively regulates fruit neck length by directly binding to the promoter of an *EXPANSIN-like A3(CsEXLA3)*, promoting cell expansion in the fruit neck. For tomato fruit shape, Bao et al. (2024) found that different microtubule-associated proteins coordinately regulate the pattern of cell division. Fruit spines (trichomes) are an important trait in cucumber. The size of their bases is one of the key traits that contribute to the different fruit appearance qualities of Chinese cucumbers and European greenhouse cucumbers (Yang et al. 2022). Zhao et al. (2024) identified the cucumber fruit spine base regulator, a C2H2-ZFP family protein CsSBS1, which is the key protein that mediates light signal to regulate spine base development in cucumber. Furthermore, they showed that CsHY5, a major transcription factor in light signaling pathways, directly binds to the promoter of *CsSBS1* and activates its expression.

Trichomes may play important roles in protecting plants from environmental stresses such as heat, low temperature, high UV radiation, and insect herbivory (Schuurink and Tissier 2020). Grandular trichomes are the site of biosynthesis and storage of specialized secondary metabolites such as flavonoids, alkaloids, terpenoids, phenylpropanoids, methyl ketones, and acyl sugars. Many of the compounds are of great importance for human consumption as pharmaceuticals, flavor and fragrance ingredients, or pesticides (Schuurink and Tissier 2020). Feng et al. (2023) identified and functionally characterized genes associated with grandular trichome organogenesis and secondary metabolism in grandular trichomes of cucumber. Transcriptomic and metabolomic analyses showed that flavonoid accumulation in cucumber grandular trichomes is positively associated with increased expression of related biosynthesis genes.

Besides the shape and smoothness of the fruit, secondary metabolites, for example anthocyanin and carotenoid, are of important value in vegetable crops. Eggplant (*Solanum melongena* L.) is a nutritious vegetable with abundant anthocyanins in its peel, but the mechanism underlying low light condition-caused low anthocyanin synthesis in fruit skins was not understood. Exogenous methyl-jasmonate (MeJA) application can effectively rescue the poor coloration of the eggplant pericarp under low light conditions. Li et al. (2024) reported an MYB transcriptional factor, SmMYB5, which promotes anthocyanin synthesis, is also regulated by light and JA signaling in eggplant pericarp. Carotenoids, especially β-carotene, are key precursors of vitamin A and have a higher antioxidant capacity. From a spontaneous cucumber yellow flesh (*yf-343*) mutant with higher carotenoid content in mature fruit flesh, Wang et al. (2023) identified the candidate gene encodes an abscisic acid (ABA) 8′-hydroxylase, which is also a key enzyme in modulating endogenous ABA homeostasis, consistent with the finding that ABA is one of the major products of the metabolic pathway of carotenoids (Sun et al. 2022). This finding highlights a promising target for gene editing to increase carotenoid content.

## Horticultural traits for cultivation and practice

Flowering occurs when plants transit from the vegetative growth to reproduction and is a key trait for crop production. Flowering is of polygenic inheritance and is affected by environment cues. Four main flowering pathways have been proposed: photoperiod, vernalization, gibberellin, and autonomous pathways. Information is integrated to the floral integrator genes, including *FLOWERING LOCUS T* (*FT*) and *SUPPRESSOR OF OVEREXPRESSION OF CO 1* (*SOC1*), which are highly conserved among most of flowering plants. In addition to gibberellin, other plant hormones including ethylene also affect flowering time. Ethylene responsive factor 070 in Pak-choi (*Brassica campestris*, *BcERF070*) is involved in flowering time control, but the detail molecular mechanism is not known. Yu et al. (2024) demonstrated that exogenous ethylene could promote *BcERF070* expressions, which reduces *LEAF*Y (*LFY*) and *FT* expression by impeding the activation of MLP328-bHLH30 complex and by promoting expression of flowering inhibitor gene *B-box 29* (*BcBBX29*).

Hypocotyl elongation is affected by light quality. Liu et al. (2024a) used a specific germplasm, Xishuangbanna cucumber to identify 2 hypocotyl elongation QTLs. Phytochrome B (PhyB) and FRIGIDA-ESSENTIAL LIKE (FEL), at the 2 target QTL intervals on chromosome 3 and chromosome 6, respectively, regulate hypocotyl elongation, suggesting that allelic variations of the 2 genes can account for hypocotyl elongation variations under low light.

Environmental factors have a significant impact on crop growth. In addition to natural factors such as temperature, water availability, and photoperiod, other components and the quality of light can have profound impacts on plant growth. Advances in technology often provide new environmental control facilities that can be utilized in crop cultivation management. Chen et al. (2024b) built several climate rooms under white- or red-rich LED lights, with or without additional far-red lights to investigate how the light quality affects flower and fruit abortion in sweet pepper. They found out that additional far-red lights increase auxin levels in shoot apices, promoting fruit abortion that was associated with reduced sucrose accumulation and lower invertase activities in flowers.

The application of ammonia borane increases endogenous hydrogen gas (H_2_) in tomato. Endogenous H_2_ homeostasis is positively correlated with later root branching. H_2_ homeostasis affects phytomelatonin homeostasis, which also affects auxin signaling network and cell-cycle (Wang et al. 2024d). Thus, ammonia borane, the new H_2_ storage material, may have applications in improving agricultural production.

High throughput phenotyping (HTP) by using multiple sensors to measure interesting traits in real time throughout the developmental stages of plants is a powerful tool in crop research and agricultural practices. Because different sensors detect different parameters or features of traits, it is really challenging to use the right sensors and to employ suitable data analyses via machine learning and deep learning to extract key features for obtaining useful information. On the other hand, user-friendly, low-cost, and stability are also considered for HTP applied in crops cultivated in open fields. The harvest index of faba bean and pea was estimated by HTP data analyzed by machine learning algorithms (Ji et al. 2024). Red-green-blue, multispectral, and thermal infrared sensors were carried by unmanned aerial vehicles to measure above ground biomass at budding, flowering, podding, and filling stages and yields as well. The harvest index was estimated by HTP data analyzed by 6 different models which performance was evaluated by normalized root-mean-square error. They concluded that combination of 2 sensors data analyzed by ensemble Bayesian model averaging model provides the most accurate harvest index estimation. This promising strategy to predict harvest index by using HTP can assist breeding selection and field management.

## Abiotic stress resistances

Cold stress, including chilling stress (0 to 12 °C) and freezing stress (below 0 °C), are detrimental to vegetable plants. The stresses seriously inhibit plant growth and reduce the quality and productivity of the produce. Tomato, being a vegetable crop derived from the tropics and subtropics, is highly vulnerable to low temperatures. Wang et al. (2024c) demonstrated that the transcription factor SlWRKY50 enhances cold tolerance in tomato by activating jasmonic acid (JA) signaling. The accumulated JA ultimately activates antioxidant enzyme genes, leading to enhanced reactive oxygen species scavenging and improved cold tolerance. However, how plants fine tune stress responses to assure optimal balance between stress tolerance and other biological processes is equally important. It is well known that when plants sense cold stimulus, the basic helix–loop–helix (bHLH) family transcriptional factor ICE1 is activated and binds to the promoter of cold-responsive genes (Hwarari et al. 2022). Lin et al. (2023) found that the Ca^2+^ sensor CaM6 negatively regulates cold tolerance in tomato plants, which is against the function of ICE1 by inhibiting its transcriptional activity. It makes sense that the weakened cold tolerance by CaM may take part in preventing exacerbated cold stress response during the late stage of the cold response in tomato plants.

Global warming is associated with the increased atmospheric CO_2_ concentrations and surface temperatures, but how elevated CO_2_ levels enhance plant resilience to heat stress is still not clear. Elevated CO_2_ generally causes an increase in glucose production, which functions both as a primary energy source and as a vital signaling molecule, regulating plant growth, defense responses, and also thermotolerance (Sharma et al. 2022). Wang et al. (2024b) reported that CO_2_-induced high glucose in the apoplast of tomato leaves binds to and triggers the endocytosis of the glucose sensor, Regulator of G protein signaling 1 (RGS1), releasing G protein α subunit (GPA1) to positively regulate thermotolerance by activating photosynthesis- and photoprotection-related genes and processes.

With the global warming, plants also must cope with drought and salt stress. A key adaptation to drought and salt stress is osmotic adjustment, accumulating proline to help reduce cellular water potential and maintain cellular functions in various ways (Shrestha et al. 2021). Liu et al. (2024b) reported how plants integrate stress signals and regulate proline synthesis in tomato. Drought and salt stress stimulate nitric oxide production, activating Δ1-pyrroline-5-carboxylate reductase (SlP5CR) by S-nitrosylation, thereby boosting both SlP5CR activity and proline synthesis during stress, increasing water use efficiency, reactive oxygen species scavenging, and sodium excretion. When subjected to water deficit, plants also exhibit a rapid surge in cytosolic Ca^2+^ levels within seconds (Aliniaeifard et al. 2020). However, how Ca^2+^ signal is perceived and relayed to the downstream osmotic signaling pathway remains largely unknown. Zhu et al. (2024) reported that 1 of the calcium-dependent protein kinase sensors, CPK27, directly interacts with and phosphorylates tonoplast sugar transporter 2 (TST2), which is enhanced by Ca^2+^ and ABA, promoting intercellular soluble sugar accumulation during drought stress.

## Biotic stress resistances

Powdery mildew (PM) is one of the most widespread and prevalent diseases that affect a wide range of crops. It is known that mutation in MILDEW RESISTANCE LOCUS O (MLO) mediated broad-spectrum, durable, and non-race specific resistance to PM, which has been utilized in breeding for decades (Kusch and Panstruga 2017). Ma et al. (2024) developed a highly efficient multiplex gene editing system in cucumber to generate a series of *Csmlo* mutants from all of the 13 family members, providing a more efficient and environmentally friendly approach to battle against this disease. *Pseudomonas syringae* is a hemibiotrophic bacterial pathogen that causes bacterial speck disease in a variety of plants, including crops such as tomato, seriously affecting crop yield and quality (Vanneste 2017). Fine-tuning of phytohormone signaling plays a crucial role in governing plant immunity against pathogen infection. Yang et al. (2024) reported that Transcription factor SlWRKY75 maintains auxin homeostasis to promote tomato defense against *P. syringae*.

Vegetable crops also utilize trichomes to protect plants against herbivorous insects and microorganisms (Zhang et al. 2021). In particular, type-IV glandular trichomes in the cultivated tomato produce acylsugars that broadly protect against arthropod herbivory. By manipulating only 2 loci, Vendemiatti et al. (2024) achieved exceptional results for a highly complex, polygenic trait, such as herbivory resistance in tomato.

## Perspectives and acknowledgements

This Focus Issue on vegetable crops has garnered attention from scientists engaged in fundamental plant biology, genetics, breeding, and biotechnology. The editorial team sincerely appreciates all authors and reviewers for their valuable contributions and efforts. While this issue may not encompass all research topics within the realm of vegetables, it covers significant progresses, novel discoveries, and new technologies. The updates and papers featured in this Focus Issue will greatly stimulate advanced research and improve agricultural practices in the field of vegetable crops.
